# Spheroid-plug model as a tool to study tumor development, angiogenesis, and heterogeneity in vivo

**DOI:** 10.1007/s13277-015-4065-z

**Published:** 2015-09-18

**Authors:** Krzysztof Szade, Monika Zukowska, Agata Szade, Guillaume Collet, Damian Kloska, Claudine Kieda, Alicja Jozkowicz, Jozef Dulak

**Affiliations:** 1 Department of Medical Biotechnology, Faculty of Biochemistry, Biophysics and Biotechnology, Jagiellonian University, Krakow, Poland; 2 Centre for Molecular Biophysics, Cell Recognition and Glycobiology, UPR4301-CNRS, Orleans, France; 3 Malopolska Centre of Biotechnology, Krakow, Poland; 4 Skaggs School of Pharmacy and Pharmaceutical Sciences, Laboratory of Bioresponsive Materials, University of California, San Diego, CA USA

**Keywords:** Cancer stem cells, Necrosis, Tumor infiltration, Tumor vascularization, B16 melanoma, Lewis lung carcinoma (LLC), Tumor complexity

## Abstract

Subcutaneous injection of the tumor cell suspension is a simple and commonly used tool for studying tumor development in vivo. However, subcutaneous models poorly resemble tumor complexity due to the fast growth not reflecting the natural course. Here, we describe an application of the new spheroid-plug model to combine the simplicity of subcutaneous injection with improved resemblance to natural tumor progression. Spheroid-plug model relies on in vitro formation of tumor spheroids, followed by injection of single tumor spheroid subcutaneously in Matrigel matrix. In spheroid-plug model, tumors grow slower in comparison to tumors formed by injection of cell suspension as assessed by 3D ultrasonography (USG) and in vivo bioluminescence measurements. The slower tumor growth rate in spheroid-plug model is accompanied by reduced necrosis. The spheroid-plug model ensures increased and more stable vascularization of tumor than classical subcutaneous tumor model as demonstrated by 3D USG Power Doppler examination. Flow cytometry analysis showed that tumors formed from spheroids have enhanced infiltration of endothelial cells as well as hematopoietic and progenitor cells with stem cell phenotype (c-Kit^+^ and Sca-1^+^). They also contain more tumor cells expressing cancer stem cell marker CXCR4. Here, we show that spheroid-plug model allows investigating efficiency of anticancer drugs. Treatment of spheroid-plug tumors with known antiangiogenic agent axitinib decreased their size and viability. The antiangiogenic activity of axitinib was higher in spheroid-plug model than in classical model. Our results indicate that spheroid-plug model imitates natural tumor growth and can become a valuable tool for cancer research.

**Electronic supplementary material:**

The online version of this article (doi:10.1007/s13277-015-4065-z) contains supplementary material, which is available to authorized users.

## Introduction

New potential antitumor drugs have to be tested in preclinical animal models before being introduced into clinical trials. Animal models have contributed to select efficient cytotoxic chemotherapeutics and helped to explain some of the mechanisms of tumor development [1, 2]. However, many drugs that showed therapeutic effects in animals failed in clinical trials [3–6]. This discrepancy indicates that preclinical animal models require improvements to better reflect complexity of tumor biology.

Among mouse tumor models, subcutaneous implants of tumor cell lines, either syngeneic or human xenografts, became most popular in basic cancer research and in drug development process [7, 8]. Subcutaneous (s.c.) models are of relatively low cost and easy to reproduce with a variety of available mouse and human tumor cell lines [7]. Nevertheless, although being simple, the s.c. models possess drawbacks [7, 8]. Tumor cells within s.c. implant do not interact with stroma of tissue of origin and tend to grow fast [7], hindering the selection of appropriate experimental end points of an experiment [7, 9], what is particularly limiting when tested drug requires long-term dosage.

These disadvantages triggered the development of novel rodent tumor models, including orthotopic models [10], carcinogen-induced tumors [11], and transgenic animal tumor models [12]. Although they resolved some problems connected with s.c. implantation of tumor cell lines, these more sophisticated approaches possess other drawbacks. Orthotopic models better reflect the tumor-stroma interactions, however, often imply advanced surgical procedures, what reduces the number of animals in experiment [7]. Carcinogen-induced models improved our understanding about processes driving cancerogenesis, but their high variability makes them rarely used in drug testing [7]. Genetic mouse models are powerful scientific tools in investigating mechanisms of tumor development, although tumors arise at various time points and are difficult to follow [8]. Additionally, genetic mouse models are costly, what all together makes them not appropriate for drug testing [7, 8]. Therefore, despite having limitations, the simple s.c. implantation of tumor cell lines is often the first choice method for investigating antitumor strategies. Thus, any improvement in making s.c. models better resembling tumor complexity while sustaining their simplicity may refine current cancer research [1].

We propose a modification of classical s.c. model. We take advantages of 3D spheroid in vitro culture of tumor cells and combine them with assets of in vivo s.c. model. In contrast to classical approach, where cells are injected as single-cell suspension, our model relies on injecting a single spheroid within a Matrigel plug. In this study, we aimed to determine if injecting the same number of tumor cells by means of classical s.c. model or spheroid-plug model has any influence on tumor development, angiogenesis, infiltration by host cells, and heterogeneity of tumor cells. We also examined both models in evaluating the efficacy of known antiangiogenic drug axitinib. Obtained results indicate that the spheroid-plug model better imitates natural tumor growth and is more suitable for long-term therapy testing.

## Materials and methods

### Ethics statement

All animal procedures and experiments were performed in accordance with national and European legislations, after approval by the First Local Ethical Committee on Animal Testing at the Jagiellonian University in Krakow (approval number 50/2012). We used C57BL/6J^OlaHsd^ mice in the study.

### Cell culture and spheroid formation

In the study, we used B16F10 melanoma and Lewis lung carcinoma (LLC) murine cell lines that were modified to stably express luciferase (luc) and green fluorescent protein (GFP). To obtain LLC-luc-GFP cell line, LLC cells were transduced with retroviral vectors produced using pMCSV-luciferase plasmid and then selected by hygromycin (25 μg/ml). Subsequently, luciferase-expressing cells were transduced by LeGO-G2 lentiviral vectors [13] (kindly provided by Prof. Boris Fehse) and double sorted for GFP^+^ cells with MoFlo cell sorter (Beckman Coulter, Brea, CA, USA). B16F10 cells were transduced with retroviral vectors produced using pBMN-eGFP-ires-luc plasmid and then double sorted for GFP^+^ cells with MoFlo cell sorter. B16F10-luc-GFP and LLC-luc-GFP cell lines were cultured in RPMI or DMEM HG medium, respectively (Lonza, Walkersville, MD, USA), supplemented with 10 % fetal bovine serum (FBS) (Biowest, Nuaillé, France) and 1 % penicillin-streptomycin (10,000 u/ml, Gibco, Invitrogen, Carlsbad, CA, USA).

Spheroids were formed by seeding 1,000 cells/well in 100 μl of medium with 0.25 % methylcellulose (R&D Systems, Minneapolis, USA) on 96-well non-adherent, rounded-bottom plate (CELLSTAR, GreinerBio-One, Frickenhausen, Germany) as described previously for endothelial cells [14]. Formation of spheroids was assessed after 48 h using phase contrast microscopy (Eclipse Ti, Nikon, Tokyo, Japan), and only spheroids with round shape were chosen for further experiments. The diameter of spheroids was approximately ~250 μm, and the number of cells in single spheroid was within 1,500–3,500 range.

### Subcutaneous injection of spheroids in Matrigel

For implantation of tumor cells, mice were anesthetized with intraperitoneal (i.p.) injection of Avertin aqueous solution (2,2,2-tribromoethanol in tert-amyl alcohol, a dose of 0.25 mg/g of body weight; Sigma-Aldrich, St. Louis, MO, USA) and shaved.

**Fig. 1:**
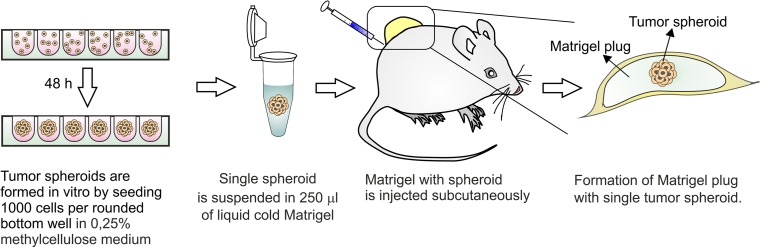
Outline of the spheroid-plug model. The spheroid-plug model relies on in vitro formation of tumor spheroid. Single spheroid is injected subcutaneously within Matrigel matrix

The spheroid-plug model relies on injection of single tumor spheroid in the Matrigel matrix. We suspend a single spheroid in cold liquid Matrigel that upon subcutaneous injection polymerizes providing a single spheroid trapped within Matrigel plug (Fig. 1). To avoid losing or disintegrating spheroid during this procedure, we used the following protocol. First, 250 μl of cold liquid growth factor-reduced Matrigel (BD Bioscience, San Jose, CA, USA) was aliquoted into precooled 1.5-ml Eppendorf tubes. Next, ~200 μl of air was drawn into the precooled 1-ml syringe (BD Plastipak, Franklin Lakes, NJ, USA) — this helps to reduce dead volume of syringe and needle. Then, half of the Matrigel volume (~125 μl) was drawn into the syringe without mounted needle. In the next step, the syringe was put into the well with the spheroid, and the spheroid was collected in ~25 μl of medium. After ensuring that spheroid is not left in the plate (binoculars are helpful), the rest of the Matrigel was drawn into the syringe, the precooled 18-G needle (Kruuse, Langeskov, Denmark) was mounted, and remaining air was removed. The Matrigel with spheroid was slowly injected subcutaneously into abdominal flank. Needle was removed after 2–3 min to let the Matrigel to polymerize and to avoid leakage. Two plugs per mice were injected. Each plug was analyzed individually. We demonstrated in vitro that transferring the spheroid according to such procedure does not affect its structure (Supplemental Fig. S1).

### Classical subcutaneous injection of tumor cells

For the classical method of subcutaneous injection of tumor cell suspension, the cells were detached from culture flasks with 0.25 % trypsin/EDTA solution and counted. For a single injection, 1,500 cells were suspended in 250 μl of liquid Matrigel matrix. The number of 1,500 cells corresponds to the lowest range of cell number in single spheroid (1,500–3,500). Therefore, injecting 1,500 cells in classical model ensures that potential differences between tumor growth models are not due to lower number of cells injected within spheroid.

### Treatment

Powder form of axitinib (Selleck Chemicals, Houston, TX 77054 USA) was dissolved in 0.5 % carboxymethyl cellulose to form homogenous solution and stored in 4 °C. Dosing started 7 days after tumor cell injection and lasted till the end of experiment. Mice from treated group were administered orally twice a day with a dose of 30 mg axitinib/kg of body weight. Similarly, control group was administered with corresponding volumes of 0.5 % carboxymethyl cellulose.

### 3D ultrasonography (3D-USG) analysis

3D-USG (VEVO 2100, MS550D transducer, VisualSonics, Toronto, ON, Canada) allowed for monitoring tumor volume along with vasculature content. Mice were shaved and anesthetized with isoflurane (1–2 % with air flow of 0.5 L/min) (Baxter, Deerfield, IL, USA). For vasculature measurements, Power Doppler mode was used with Doppler gain 35 dB, dynamic range 35 dB, and wall filter in medium mode. Measurements were conducted in three time points: 1, 7, and 14 days after the injection of tumor cells. The vascularization was expressed as percentage of vascularization, what represents the percentage of pixels in a given area with positive Power Doppler signal. Results were analyzed in 3D mode using VEVO 2100 Software.

### In vivo detection of bioluminescence

Tests were conducted along with 3D-USG analysis in the same three time points, using In Vivo Imaging System (IVIS) Lumina (PerkinElmer, Waltham, MA, USA). Twenty minutes before imaging, mice were injected i.p. with 0.2 ml luciferin (15 mg/ml, Promega GmbH, Mannheim, Germany). Five minutes before measurement, animals were anesthetized with isoflurane. Exposure time for bioluminescence measurements was 60 s with medium binning mode. In case of oversaturation of detected signal, the time was shortened. Obtained data are presented as average radiance [photon/s/cm^2^/sr]. This unit is called further as radiance units (RU).

For detection of metastasis in organs, mice were injected i.p. with luciferin 15 min before sacrifice. Next, lungs, liver, and intestines were excised, and bioluminescence was measured with exposure of 60 s, binning mode. Mice with positive bioluminescent signal in any of the organs were counted as possessing metastases.

### Flow cytometry analysis

Tumor plugs were dissected and divided into two parts: one for flow cytometry and second for histological analysis. For flow cytometry analysis, plug was chopped using a scalpel, placed in 2-ml Eppendorf tube, and digested in 0.5 ml of enzyme mix (37 °C, 1 h) which consisted of Liberase TM 3 U/ml (Roche, Indianapolis, IN, USA), hyaluronidase 25 μg/ml (Sigma-Aldrich, St. Louis, MO, USA), DNAse 25 μg/ml (Roche, Indianapolis, IN, USA), and dispase 3 U/ml (Sigma-Aldrich, St. Louis, MO, USA). After 1 h, 0.5 ml of FBS was added, and samples were placed on ice to stop the reaction. Cell suspension was filtered through 70-μm strainer (BD Bioscience, San Jose, CA, USA), centrifuged (600×*g*, 10 min, 4 °C), and stained in 2 % FBS in PBS for 20 min on ice with antibodies listed in Supplemental Fig. S2. Flow cytometry was performed using BD LSR II (BD Bioscience, San Jose, CA, USA) with excitation lasers 405, 488, and 633 nm. Results were analyzed using FACS DIVA software (BD Bioscience, San Jose, CA, USA) and FlowJo software (Tree Star, USA).

### Histological analysis

Dissected tumor plugs were kept overnight in freshly prepared formalin solution and dehydrated in tissue processor (Shandon Excelsior ES, Thermo Scientific, Kalamazoo, MI, USA) using reagents provided by DiaPath (DiaPath, Martinengo, Italy). Dehydrated plugs were embedded in paraffin, cut into 4-μm-thick sections on microtome (HM 355S, Thermo Scientific, Kalamazoo, MI, USA), and stained with hematoxylin and eosin (Sigma-Aldrich, St. Louis, MO, USA) in Varistain Gemini automated slide stainer (Thermo Scientific, Kalamazoo, MI, USA), according to a standard protocol. Prepared sections were analyzed under a microscope (Eclipse Ti, Nikon, Tokyo, Japan). Pictures were taken using NIS-Elements BR software. Content of necrotic tissue was assessed using AxioVision 40 V 4.8.2 software (Carl Zeiss Microscopy, Jena, Germany).

### Statistical analysis

Statistical analysis and presentation of data were performed with the use of GraphPad Prism 6 software (GraphPad Software, San Diego, CA, USA). Obtained results are presented as means ± SEM. Statistical significance was analyzed using non-paired Mann–Whitney test. If more than two groups of data were analyzed with more than one variable, two-way ANOVA with Bonferroni post-test was used, preceded by logarithmic transformation in the case of log-normal distribution of values.

## Results

### Tumor growth is slower in spheroid-plug model

**Fig. 2:**
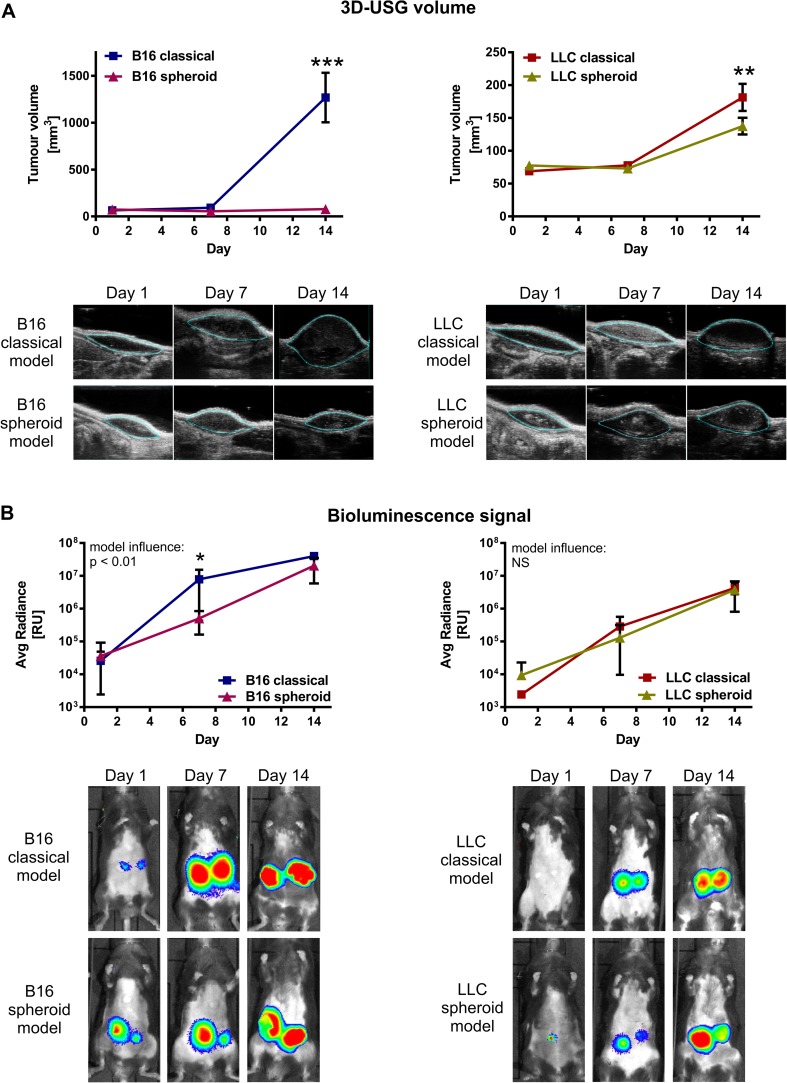
Different kinetics of growth in spheroid-plug model and classical model. **a** Tumor volumes measured by 3D-USG in spheroid-plug and classical model. **b** In vivo bioluminescence signal (IVIS) from spheroid-plug and classical model tumors. Two-way ANOVA with Bonferroni post-test (*p < 0.05, **p < 0.01, ***p < 0.001) between models in a given day, *n* = 6–12

First, we compared the kinetics of tumor growth in classical and spheroid-plug model using noninvasive 3D-USG. Size of B16 tumors injected by classical method increased logarithmically (14.37 ± 4.51-fold increase at day 14) (Fig. 2a). In contrast, in spheroid-plug model, B16 tumor plugs did not significantly change their size (1.07 ± 0.12-fold increase) (Fig. 2a). Consistently, LLC tumors injected by spheroid-plug model were also smaller than tumors injected classically (1.82 ± 0.20- vs. 2.69 ± 0.39-fold increase at day 14, *p* < 0.01); however, the difference between the models was much less pronounced than in B16 tumors (Fig. 2a).

In addition to 3D-USG technique, we compared kinetics of tumor growth by in vivo detection of bioluminescence (IVIS). Two-way ANOVA indicates that model applied for implantation of B16 cells influences the tumor growth (*p* < 0.01), with significantly faster increase in luminescence in case of classical method (Fig. 2b). However, at day 14, the IVIS signal intensity from B16 tumors in spheroid-plug model was much higher than on the day 1 and similar to that in classical model (2.04 ± 0.46 × 10^7^ vs. 3.99 ± 0.23 × 10^7^ at day 14, NS) (Fig. 2b). Thus, at day 14, the difference between models in tumor volumes was notably higher (15.16-fold, Fig. 2a) than the difference in IVIS signal (1.96-fold, Fig. 2b). Accordingly, in the case of LLC tumors, we did not notice differences in IVIS signal (Fig. 2b) between models (4.26 ± 0.53 × 10^6^ vs. 3.79 ± 0.86 × 10^6^), despite small but statistically significant difference in LLC tumor volumes (Fig. 2a).

We concluded that spheroid-plug tumors grew slower than tumors formed in classical model. However, measurements of tumor volumes by 3D-USG were not paralleled by detection of tumor cell-derived luminescence detected by IVIS.

### Tumors in spheroid model are less necrotic

**Fig. 3:**
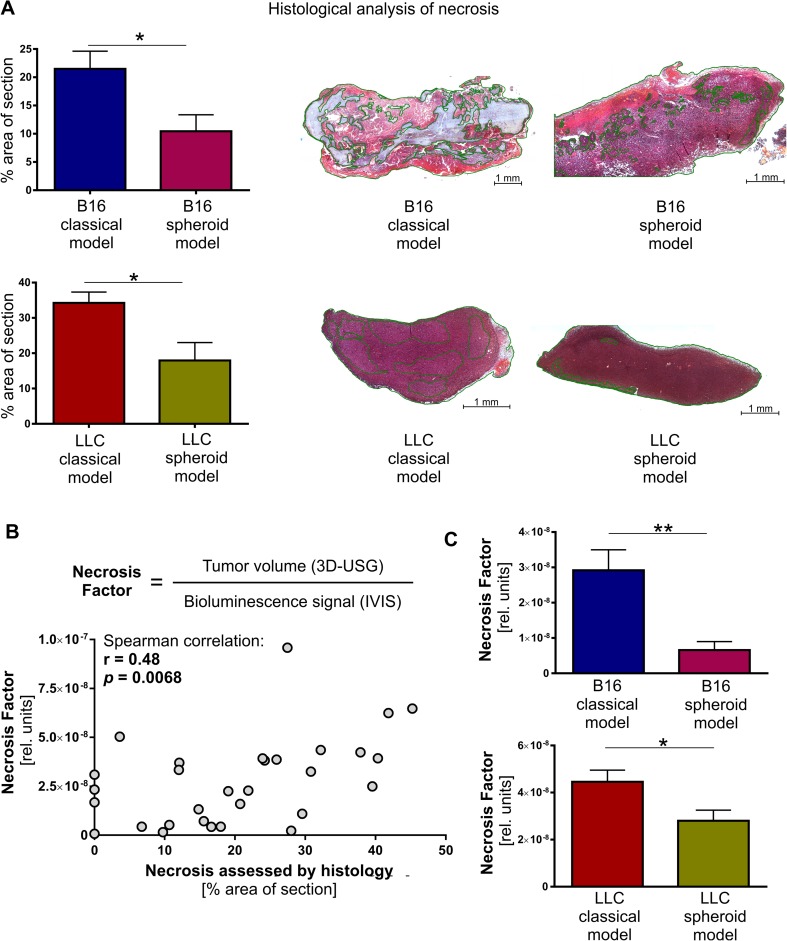
Lower necrotic content in spheroid-plug tumors. **a** H&E staining of necrotic areas within tumors from spheroid-plug model. **b** Necrosis factor (NF) estimation of necrotic content in tumors by noninvasive in vivo imaging and NF correlation with necrotic content in tumors assessed by H&E staining. **c** NF in spheroid-plug model in B16 and LLC tumors. *p < 0.05, **p < 0.01, Mann–Whitney test

In the next step, we investigated possible reasons for the discrepancy between tumor volumes and IVIS signal intensities. Because only viable cells are able to express luciferase and produce detectable bioluminescence signal, the inconsistency between tumor volumes and IVIS measurements may indicate the presence of necrotic, not viable areas within tumor. Indeed, histological analysis using hematoxylin and eosin staining confirmed that B16 and LLC tumors from spheroid-plug model had smaller area of necrotic tissue than tumors from classical method (B16 10.46 ± 2.91 vs. 21.49 ± 3.13 %, *p* < 0.05; LLC 17.95 ± 5.06 vs. 34.26 ± 3.06 %, *p* < 0.05) (Fig. 3a).

Given that variable necrosis content results in discrepancy between tumor volumes and IVIS signal, we proposed that a ratio of tumor volume (measured, e.g., using 3D-USG) to the IVIS signal intensity from the tumor (Fig. 3b) may be used to evaluate necrosis content within tumors in quantitative and noninvasive manner. We demonstrated that this parameter, named here the necrosis factor (NF), correlates significantly with necrosis content in all analyzed tumors estimated by histology (*r* = 0.48, *p* < 0.0068, Spearman rang correlation) (Fig. 3b). The calculated NF showed significant, consistent with histological analysis, differences between models: its value was lower in spheroid-plug model, both in B16 (*p* < 0.01) and LLC tumors (*p* < 0.05) (Fig. 3c).

Altogether, the spheroid-plug tumors were less necrotic. Our analysis showed also that in vivo imaging could be used to calculate necrosis factor as noninvasive indicator of tumor necrosis.

### Spheroid-plug model ensures stable vascularization of tumors

**Fig. 4:**
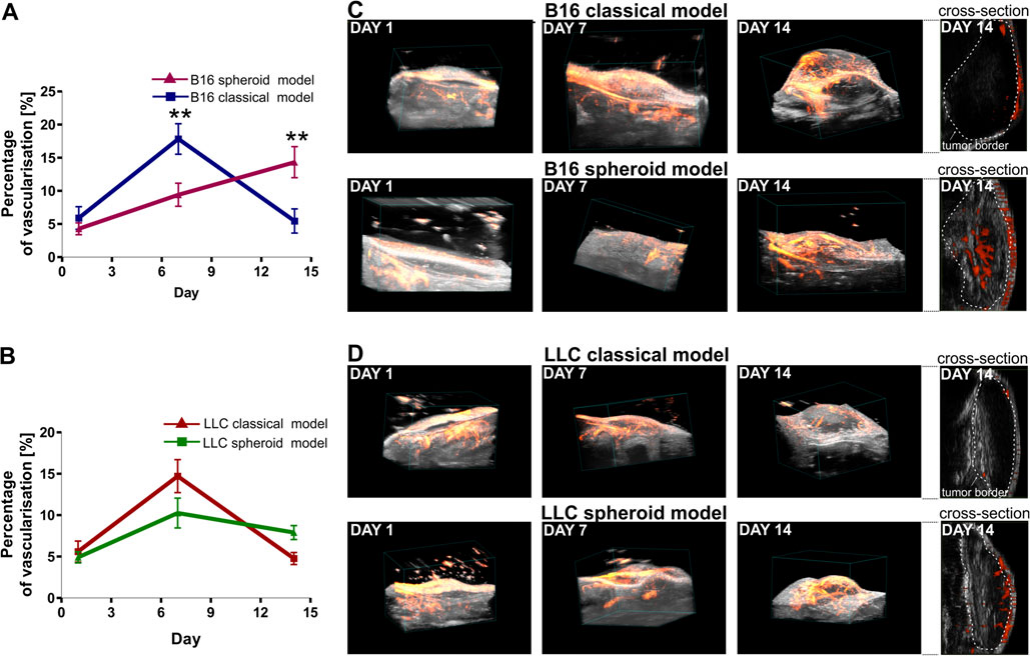
Development of tumor vasculature in spheroid-plug and classical model assessed by Doppler 3D-USG analysis. **a** Vascularization in B16 spheroid-plug and classical model tumors, observed at days 1, 7, and 14. **b** Vascularization in LLC tumors observed at days 1, 7, and 14. **c**, **d** Visualization of vessels in classical and spheroid-plug model. Two-way ANOVA with Bonferroni post-test (**p < 0.01) between models in a given day

Necrotic areas of tumors are often the consequence of inadequate and nonfunctional tumor vasculature [15, 16]. We monitored development of vasculature within tumors in vivo (days 1, 7, and 14 after tumor injection) using 3D-USG with perfusion analysis. Performed measurements indicated different kinetics of tumor angiogenesis between classical and spheroid-plug model in B16 tumors. While at day 7 we observed a higher perfusion in classical model (17.84 ± 2.31 vs. 9.42 ± 1.74 %, *p* < 0.01) (Fig. 4a), at day 14, it decreased and was lower than in spheroid model (5.45 ± 1.82 vs. 14.33 ± 2.35 %, *p* < 0.01) (Fig. 4a). The observed pattern — slower development of vasculature at day 7 (14.71 ± 1.99 vs. 10.93 ± 1.82 %) but higher vascularization at day 14 (4.77 ± 0.73 vs. 7.90 ± 0.84 %) in spheroid-plug model compared to classical model — was also visible in LLC tumors; however, the differences did not reach statistical significance (Fig. 4b).

Furthermore, we noticed the difference in vessel localization within tumors (Fig. 4c, d). At day 14, in classical model, vessels were visible only near the tumor borders, whereas in spheroid-plug model, vessels penetrated the tumor core, as shown by visualization of 3D-USG analysis (Fig. 4c, d).

### Infiltration of cells with progenitor phenotype is increased in spheroid-plug model

**Fig. 5:**
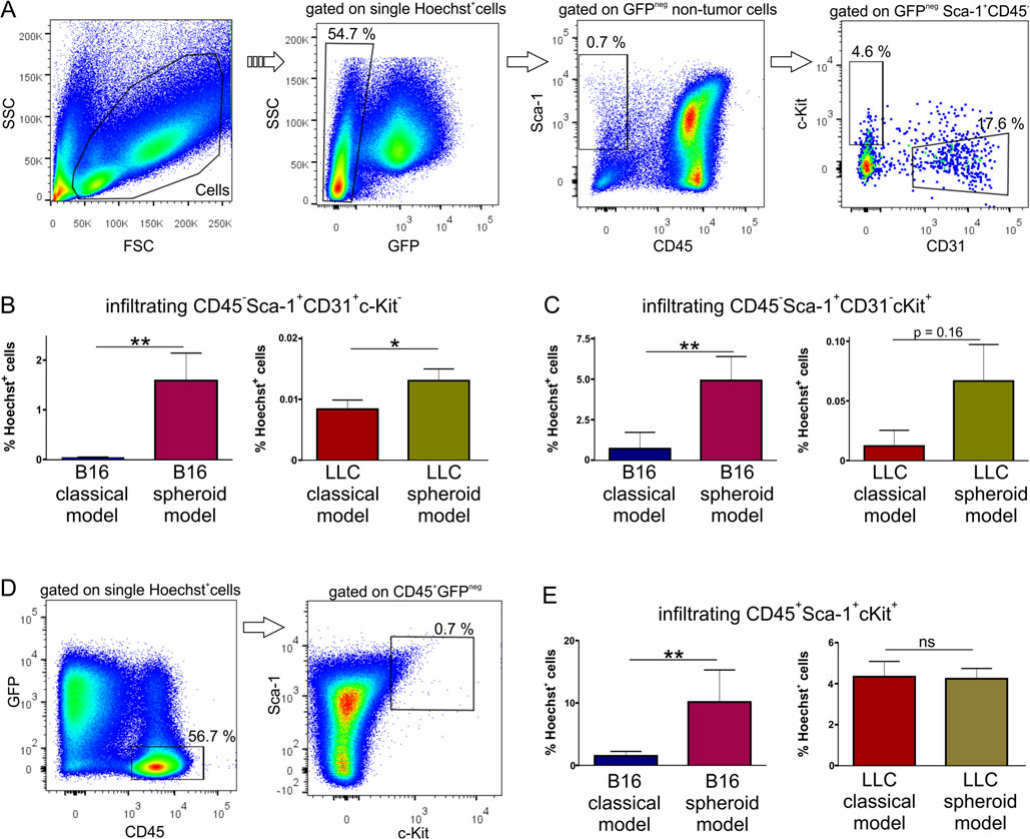
Infiltration of tumors in spheroid-plug and classical model. **a** Representative flow cytometry analysis of cells infiltrating tumors. **b** Infiltration of endothelial cells in tumors from spheroid-plug and classical model. **c** Content of CD45^−^Sca^+^CD31^−^c-Kit^+^ non-hematopoietic progenitor cells in spheroid-plug and classical model tumors. **d** Representative flow cytometry analysis of hematopoietic cells infiltrating tumors. **e** Infiltration of CD45^+^cKit^+^Sca-1^+^ hematopoietic cells within tumors. *p < 0.05, **p < 0.01, Mann–Whitney test

Infiltrating host cells may drive tumor angiogenesis [17, 18]. As spheroid-plug tumors were more vascularized, we checked if increased vascularization correlates with presence of other host cells. We took advantage of GFP-expressing tumor cells, what enabled us to distinguish GFP^+^ tumor cells from GFP^−^ infiltrating cells by using flow cytometry (Fig. 5a).

First, we checked the content of endothelial cells. The total number of endothelial cells with CD45^−^Sca-1^+^CD31^+^c-Kit^−^ phenotype (Fig. 5a) was significantly higher in the case of spheroid-plug model than in classical model, both in B16 (1.52 ± 0.55 vs. 0.039 ± 0.009 %, *p* < 0.01) and LLC (0.13 ± 0.061 vs. 0.083 ± 0.015 %, *p* < 0.05) tumors (Fig. 5b).

Next, among infiltrating cells, we distinguished population with non-hematopoietic progenitor cell phenotype CD45^−^Sca-1^+^CD31^−^cKit^+^ (Fig. 5a). This population was significantly increased in spheroid-plug model compared to the classical model in case of B16 tumors (4.92 ± 1.48 vs. 0.71 ± 0.36 %, *p* < 0.01), and similar tendency was observed in LLC tumors (0.067 ± 0.03 vs. 0.012 ± 0.004 %, *p* = 0.16) (Fig. 5c).

Apart from endothelial and mesenchymal cells [19–21], various hematopoietic progenitors may also regulate angiogenesis [22, 20, 21]. Indeed, we found that cells with hematopoietic progenitor phenotype CD45^+^Sca-1^+^c-Kit^+^ infiltrated tumors (Fig. 5d). These cells were more abundant in spheroid-plug model than in classical model in B16 tumors (10.16 ± 5.13 vs. 1.51 ± 0.71 %, *p* < 0.01) (Fig. 5e). However, in the case of LLC, there were no differences in the content of hematopoietic progenitors between tested models (Fig. 5e).

Taken together, we observed that in B16 tumors, the augmented angiogenesis in spheroid-plug model correlated not only with higher number of endothelial cells but also with increased infiltration of cells expressing progenitor markers (c-Kit and Sca-1). Such relationship seems to be tumor type specific, as it was not observed in tumors formed by LLC cells.

### Spheroid-plug model increases the number of cells with cancer stem cell phenotype

In the next step, we compared heterogeneity of tumor cells between spheroid-plug and classical models. Given that in vitro 3D spheroid culture facilitates the acquisition of cancer stem cell properties [23–25], we wondered if there were more tumor cells with cancer stem cell phenotype in tested spheroid-plug model. To verify this hypothesis, we analyzed expression of several markers on GFP^+^ tumor cells that were previously thought to characterize cancer stem cells: c-Kit [26], Sca-1 [27], CD133 [28–30], CD49f [31], and CXCR4 [30, 32, 33].

**Fig. 6:**
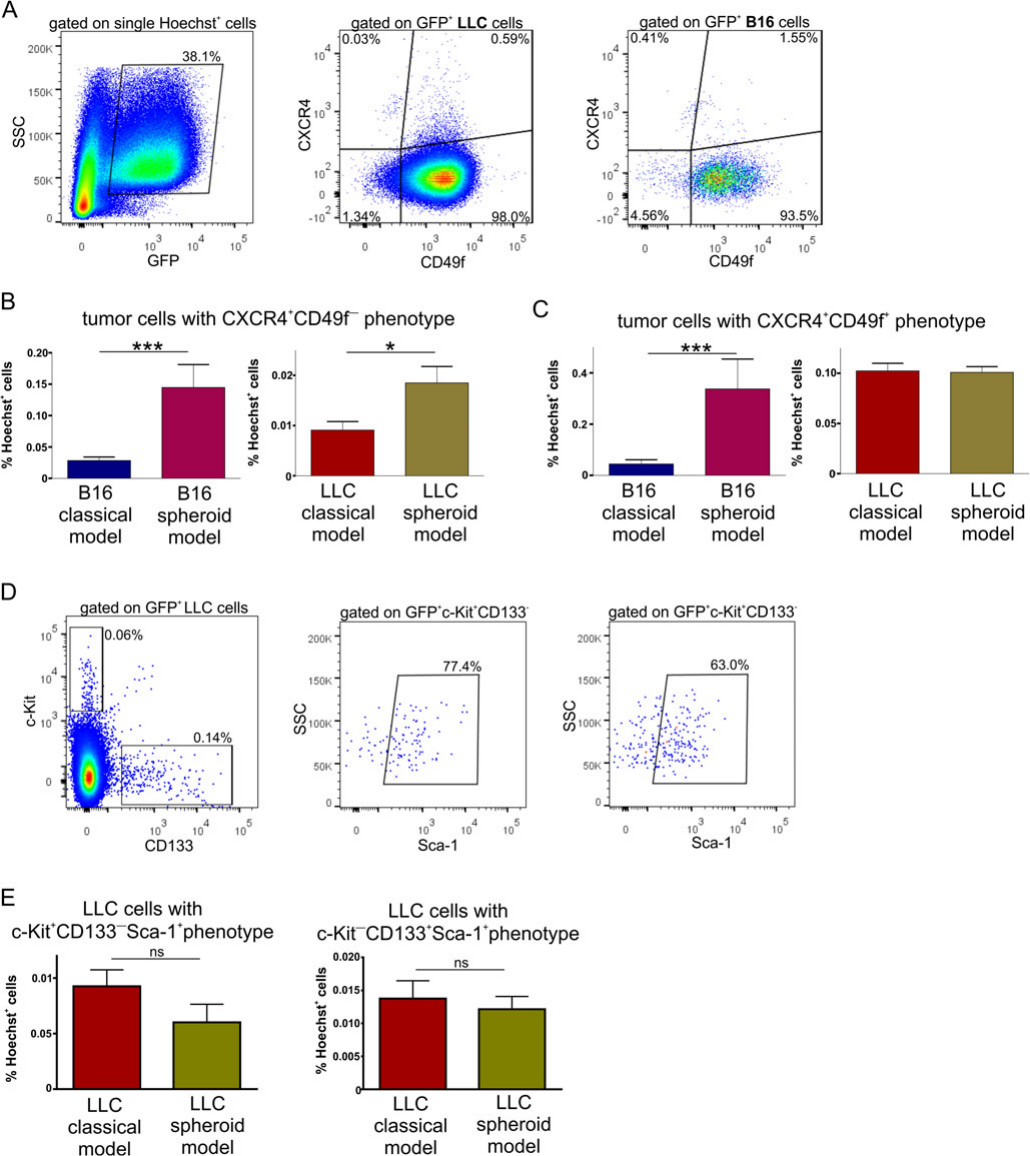
Analysis of stem cell markers expression in tumors from spheroid-plug and classical model. **a** Representative flow cytometry analysis of CXCR4 and CD49f expression on tumor cells. **b** The CXCR4^+^CD49^−^ subpopulation in spheroid-plug model in both B16 and LLC tumors. **c** The CXCR4^+^CD49^+^ subpopulation—in B16 tumors and LLC tumors. **d**, **e** c-Kit^+^CD133^−^Sca-1^+^ and c-Kit^−^CD133^+^Sca-1^+^ population among LLC tumors, **p < 0.01 Mann–Whitney test

The obtained results revealed that most of the LLC and B16 tumor cells expressed CD49f, what suggests that this antigen alone did not select unique cancer stem cells in investigated models (Fig. 6a). Nevertheless, we used CD49f together with CXCR4 to analyze heterogeneity of tumor cells (Fig. 6a). We detected a minor population expressing CXCR4 among LLC and B16 cells, both within CD49f^+^ and CD49f^−^ subsets (Fig. 6a). The number of CXCR4^+^CD49f^−^ tumor cells was higher in spheroid-plug model than in classical model either in B16 or LLC tumors (Fig. 6b), while CXCR4^+^CD49f^+^ population was more abundant in spheroid-plug model only in B16 tumors (Fig. 6c). Expression of c-Kit, Sca-1, or CD133 did not distinguish separate populations in B16 tumors. Differently, in LLC tumors, we found c-Kit^+^CD133^−^ and c-Kit^−^CD133^+^ subpopulations that expressed also Sca-1 (Fig. 6d); however, there were no differences in content of these subpopulations between tested models (Fig. 6e).

Finally, we tried to explain why some of the described populations expressing stem cell markers were changed in spheroid-plug model, while other did not differ. One possibility is that in vitro spheroid culture itself induces cells with cancer stem cell phenotype, which then persist during in vivo tumor development. Flow cytometry analysis confirmed that at least part of populations expressing stem cell markers were induced already by 3D culture conditions in vitro. We observed that spheroid culture increased frequency of CXCR4^+^CD49f^+^ B16 cells (Supplemental Fig. S3A) consistently with changes observed in vivo in spheroid-plug model (Fig. 6c). Similarly, spheroid culture led to increased number of CXCR4^+^CD49f^−^ LLC cells (Supplemental Fig. S3B), in concert to cell profile observed in vivo in spheroid-plug model (Fig. 6b).

In contrast, in in vitro spheroid culture, we did not detect CXCR4^+^CD49f^−^ population in B16 cells as well as CXCR4^+^CD49f^+^ and c-Kit^+^CD133^−^Sca-1^+^ subsets in LLC cells (data not shown). Moreover, c-Kit^−^CD133^+^Sca-1^+^ population was induced in LLC spheroids in vitro (Supplemental Fig. S3C), what was not observed in spheroid-plug LLC tumors (Fig. 6e). Thus, the altered frequency of these populations in vivo in spheroid-plug model cannot be explained by the changes caused by spheroid formation in vitro.

### Spheroid-plug model is suitable for testing the efficacy of antiangiogenic drugs

**Fig. 7:**
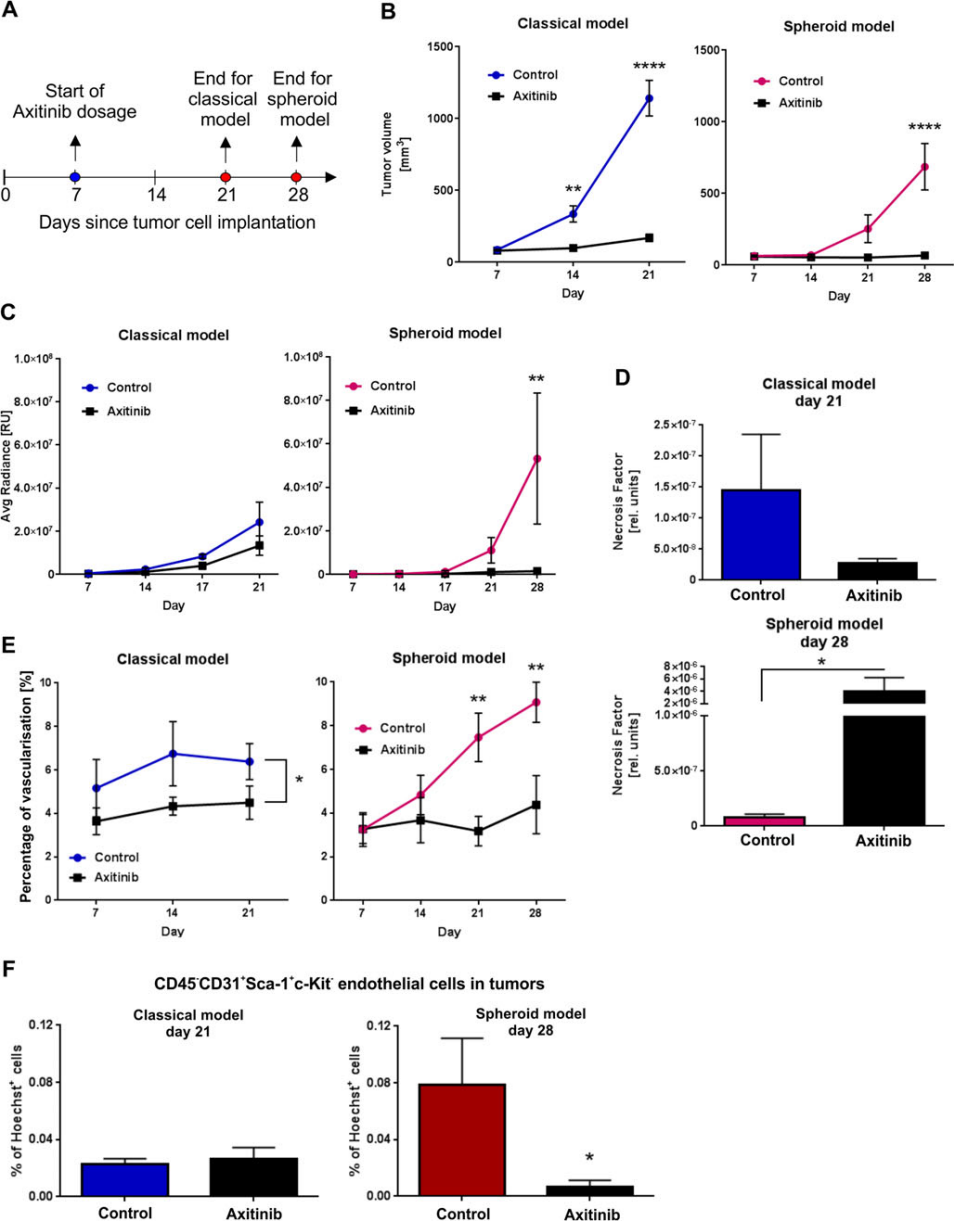
Accuracy of classical and spheroid-plug model in predicting efficiency of tumor treatment. **a** Scheme of experiment. **b** Influence of axitinib treatment on tumor volumes in classical and spheroid-plug model. Two-way ANOVA with Bonferroni post-test (**p < 0.01, ****p < 0.0001) between groups in a given day, *n* = 7–12. **c** In vivo bioluminescence signal (IVIS) from treated and untreated tumors in both models. Two-way ANOVA with Bonferroni post-test (**p < 0.01) between groups in a given day, *n* = 9–12. **d** NF in classical and spheroid model after treatment. Mann–Whitney test: classical model, *p* = 0.573; spheroid-plug model, *p < 0.05; *n* = 8–12. **e** Changes in vascularization of classical and spheroid-plug tumors after axitinib. Classical model: two-way ANOVA, *p < 0.05 between groups, *n* = 8–12. Spheroid model: two-way ANOVA with Bonferroni post-test (** *p* < 0.01) between groups in a given day, *n* = 7–12. **f** Frequency of endothelial cells in tumors at the end of experiments assessed by flow cytometry. Mann–Whitney test: *p < 0.05, *n* = 8–12

To compare the efficacy of known antiangiogenic drug in classical and spheroid-plug model, we chose axitinib which is a new type of small molecule tyrosine kinase inhibitor [34] with evidenced effectiveness in B16 tumor model [35, 36]. The treatment with axitinib was started 7 days after injection of tumors (Fig. 7a).

Firstly, we compared B16 tumor growth kinetics between treated and control groups in both models with the use of 3D-USG. Again, we could observe fast logarithmic growth of tumor volume in classical model (13.89 ± 1.68-fold increase at day 21 comparing to day 7 in control group) (Fig. 7b). In mice dosed with axitinib changes in tumor volume were smaller (2.08 ± 0.31-fold increase) (Fig. 7b). The fast growth of tumors in classical model forced us to terminate experiment at day 21.

In comparison, control tumors from spheroid-plug model grew slower (2.86 ± 0.92-fold increase at day 21 when comparing to day 7), and we were able to prolong experiment till day 28 (Fig. 7b). Similarly to classical model, we observed significant difference in tumor volumes between control and treated groups at the end of experiment (684.6 ± 162.2 mm^3^ in control group vs. 64.34 ± 13.56 mm^3^ in axitinib group, *p* < 0.0001) (Fig. 7b).

We also monitored tumor development by IVIS. Although axitinib significantly decreased the size of tumors at the end of the experiment in both models, there was no significant difference in IVIS signal in classical model (Fig. 7c).

On the other hand, in spheroid-plug model, the axitinib treatment not only decreased tumor size but also resulted in substantial differences in IVIS signal between treated and non-treated groups (37.01-fold lower signal in axitinib group at day 28, *p* < 0.01) (Fig. 7c). Additionally, calculated necrosis factor values showed that axitinib significantly influenced necrosis content only in spheroid-plug model, whereas it had no effect on tumors in classical model (Fig. 7d).

Axitinib is an antiangiogenic drug; therefore, we evaluated tumor vascularization as an important parameter reflecting the drug efficiency. Measurements performed using 3D-USG with perfusion analysis revealed different effect of axitinib on tumor vasculature in examined models. Axitinib treatment had bigger effect on tumors in spheroid-plug approach: treated tumors were significantly less vascularized than those from control group (3.18 ± 0.68 vs. 7.47 ± 1.11 % at day 21, *p* < 0.01; 4.38 ± 1.33 vs. 9.08 ± 0.92 % at day 28, *p* < 0.01) (Fig. 7e). In contrast, differences between groups in classical model were smaller and did not reach statistical significance at the end of experiment (4.5 ± 0.77 % in treated group vs. 6.38 ± 0.83 % in control at day 21, NS) (Fig. 7e). Consistently, we observed significantly less endothelial cells (defined by flow cytometry as CD45^−^CD31^+^Sca-1^+^c-Kit^−^) after treatment with axitinib in spheroid-plug model, what was not visible in classical model (Fig. 7f).

**Fig. 8:**
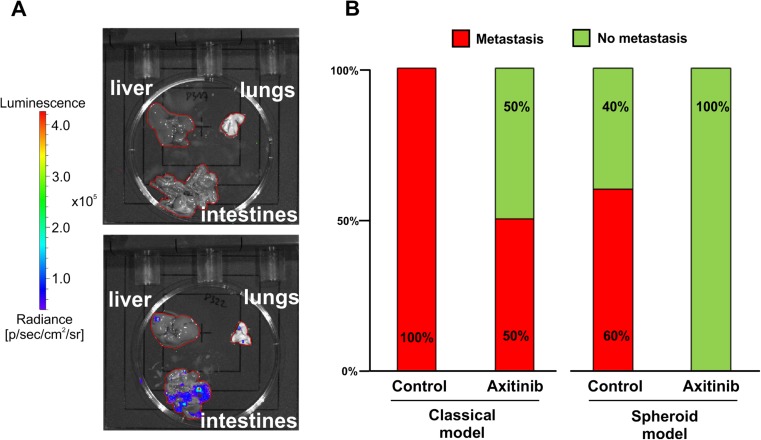
Metastasis occurrence in classical and spheroid-plug model after axitinib treatment. **a** Organs without metastasis (*upper*) and showing positive bioluminescent signal (*lower*) and **b** comparison of metastasis occurrence in both models after axitinib treatment, *n* = 4–6

Moreover, we also examined axitinib influence on metastasis occurrence in both models (Fig. 8a). We were able to observe that 60 % of spheroids administered subcutaneously in the control group formed metastasis after 28 days. Axitinib treatment completely inhibited that process in spheroid-plug model (Fig. 8b). In classical model, all mice from control group developed metastasis already after 21 days, but treatment with axitinib showed only 50 % efficacy in metastasis inhibition (Fig. 8b).

## Discussion

In the present study, we describe the novel in vivo tumor growth model based on subcutaneous delivery of cancer cells in the form of single spheroid. Although the interest in tumor spheroids began in the 70s [37], till now, only few reports described studies on their in vivo application [38, 39]. These previous works concerned intraperitoneal injections of multiple spheroids and were limited to the characterization of dissected spheroids [38, 39]. In contrast, we proposed subcutaneous injection of single spheroid within Matrigel to analyze tumor growth, vascularization, host cell infiltration, and tumor cell heterogeneity.

**Fig. 9:**
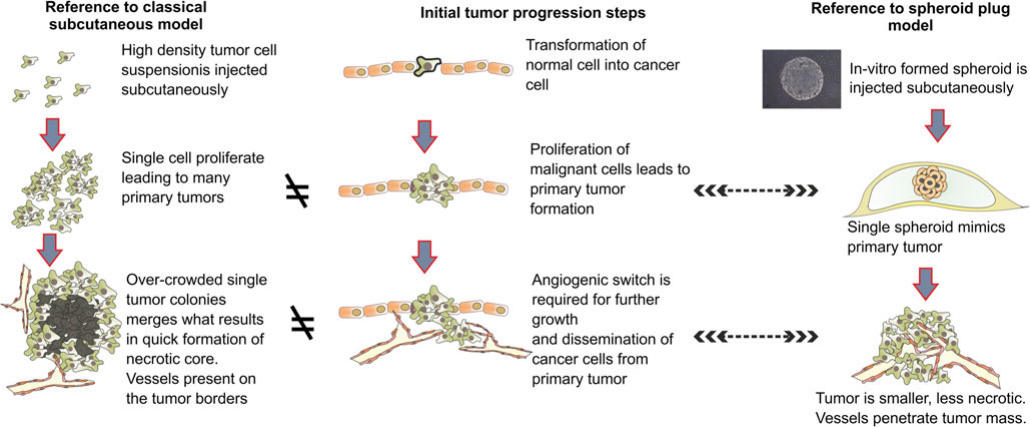
Rationale for the spheroid-plug model

The rationale for injection of single spheroid instead of suspension of single cells is that single spheroid better imitates the initial stages of tumor progression (Fig. 9). The tumor usually develops from one or few transformed cells which form primary tumor lesion [40]. In spheroid-plug model, this primary tumor lesion is mimicked by injection of a single spheroid in Matrigel suspension, comprising 1,500–3,500 cells (Fig. 9). Oppositely, in classical model, each individual injected cell may form the tumor lesions, which subsequently merging together form a bigger tumor mass. Given that number of injected cells in classical model reaches millions in some protocols [9], this classical subcutaneous model is significantly distinct from current knowledge about initial stages of tumor development. Therefore, we proposed a spheroid-plug model as a modification of classical subcutaneous injection of tumor cells to more accurately reflect mechanisms of tumor growth.

To our best knowledge, this is the first attempt that relies on in vitro cultured tumor spheroids combined with in vivo subcutaneous implantation. Our results indicate that the method which is used to subcutaneously inject cancer cells — either classical as cell suspension or as spheroid — had impact on several aspects of tumor development.

First, we showed that growth rate of tumors is slower in the spheroid-plug model compared to the classical one (Fig. 2a). The fast growth of tumors after subcutaneous implantation is regarded as artificial and often limits the proper selection of experiment end points [7, 9]. Despite injecting small number of cells (1,500) in suspension, the tumors still grew artificially fast, indicating that this might be an intrinsic feature of classical subcutaneous method. Therefore, the slower growth of spheroid-derived tumors in Matrigel plug may at least partially surmount this limitation of subcutaneous models. This ability to prolong the course of study is particularly important when the effects of tested drugs or therapies require longer time to occur. An example of such therapies may be immunotherapies which need time to stimulate immunological responses [41, 42].

Not only is the tumor growth rate affected in spheroid-plug model but also the necrosis content differs. The smaller size of tumors correlates with smaller necrosis in spheroid-plug model comparing to classical model (Fig. 3). Both tumor size and necrosis content correlate with stage and prognosis of many tumors [43, 44]. Taken together, smaller size and lower necrosis content indicate that spheroid-plug tumors mimicked less advanced stage than tumors from classical models.

Next, we observed that development of tumor vascularization follows distinct patterns in classical and spheroid-plug model, especially in B16 tumors (Fig. 4a, b). In classical model, vascularization of tumors reached highest values already at day 7 and then significantly dropped at day 14. Oppositely, in the spheroid-plug model, tumor angiogenesis progressed slower and the vascularization at day 7 was lower than in classical model but sustained or even increased (in B16 tumors) at day 14.

Tumor angiogenesis is an important target of currently being developed anticancer strategies [45]. However, many studies demonstrating efficacy of antiangiogenic drugs in animal subcutaneous models were not confirmed in clinical trials [46, 47]. Our analysis demonstrated that tumor vascularization develops differently in spheroid-plug model comparing to classical subcutaneous implantation. Given that the tumor angiogenesis differs between the models, it is justified to suppose that outcome of antiangiogenic drugs tests may depend on model used in such studies.

Along with targeting tumor angiogenesis, new anticancer treatments focus also on regulating the infiltration of host cells [18, 20]. Different progenitors of non-hematopoietic and hematopoietic origin infiltrate tumor and may facilitate tumor growth [19, 21]. On the other hand, the potential to infiltrate tumors may be also used as a tool to deliver therapeutic genes into tumors [48–51]. We tested if the spheroid-plug model could be used to study tumor infiltration by the host cells. B16 tumors from spheroid-plug model were more infiltrated by hematopoietic and non-hematopoietic cells with progenitor phenotype. Increased infiltration of tumors by progenitor cells makes the spheroid-plug model a valuable tool to study this phenomenon.

Next, we addressed the aspects of heterogeneity of tumor cells in spheroid-plug model with focus on cells with cancer stem cell phenotype. We evaluated expression of several markers shown before to characterize cancer stem cells. Consistently with previous reports [33, 52], we were able to distinguish among both LLC and B16 tumor minor subpopulations with CXCR4 expression that is thought to characterize cancer stem cells. Additionally, we showed that CXCR4^+^ fraction could be further divided based on CD49f expression, what to our best knowledge was not shown before. However, if expression or lack of expression of CD49f enriches the CXCR4^+^ population for functional cancer stem cells needs to be further elucidated.

It is known that in vitro culture of tumor cells in form of 3D spheroids changes their phenotype and increases heterogeneity among tumor cells, including induction of stem cell phenotype [23, 24, 53, 54]. Indeed, we showed that some of the cancer stem cell populations upregulated in vivo in spheroid-plug model tumors are already induced by in vitro spheroid culture (CXCR4^+^CD49f^−^ in LLC and CXCR4+CD49f^+^ in B16, Supplemental Fig. S3). Nevertheless, other tumor populations with stem cell markers (CXCR4^+^CD49f^−^ in B16 and CXCR4^+^CD49f^+^, c-Kit^−^CD133^+^Sca-1^+^, c-Kit^+^CD133^−^Sca-1^+^ in LLC) detected in vivo in spheroid-plug model cannot be simply linked with induction of these populations by in vitro 3D conditions, as they were either not detected or were differentially regulated by in vitro spheroid culture. Therefore, increased heterogeneity among tumor cells observed in spheroid-plug model at least partially seems to be a result of different characteristic of tumor growth in vivo. This makes spheroid-plug model a valuable tool to study tumor heterogeneity and cancer stem cells.

In the present study, we used two different cell lines: B16F10 and LLC. Spheroid-plug model could be applied to other tumor cell lines as long as they are capable of forming spheroids in vitro. It should be tested if the spheroids are compact enough to sustain their structure during injection. We evaluated other cancer cells lines and found that tumor cell lines form spheroids easily (e.g., SMS-CTR and CW9019 rhabdomyosarcoma, data not shown). However, there were tumor cell lines which formed clumps of cells instead of solid spheroids (e.g., squamous cell carcinoma SSC-VII). This limitation can be overcome by mixing tumor cells with other cells (we checked fibroblasts or cultured mesenchymal stem cells, data not shown), as suggested by Augustin and co-workers [55]. Such co-spheroids are more compact and could be injected subcutaneously with the described method.

Though the tumor cell line forms spheroids, the difference between spheroid-plug model and classical model may be stronger or weaker depending on given tumor cell line. We suppose that higher growth rate as well as dependence on vascularization and cancer stem cells may result in bigger impact of spheroid-plug model on tumor growth in comparison to classical method. In our work, the differences between models are best visible in case of B16 cells, while in case of LLC they were less evident and sometimes reached only borderline statistical significance. This could be linked with much higher tumor growth rate of B16 tumors in comparison to LLC tumors (1,268 ± 263.4 vs. 181.3 ± 20.7 mm^3^, *p* < 0.001, at day 14 in classical models, Fig. 2a). Nevertheless, the course of changes associated with spheroid-plug model was the same in B16 and LLC tumors.

Additionally, we postulated that simultaneous measurement of tumor volume with IVIS analysis may be used to estimate tumor necrosis (Fig. 3b), by calculating NF. Although the proposed necrosis factor cannot replace histological analysis, it may be applied as an approximate determinant of tumor necrosis that can be monitored by noninvasive in vivo imaging.

Finally, to validate the proposed spheroid-plug model as a tool for drug testing, we compared the efficacy of a known antiangiogenic agent in classical and spheroid-plug model. To this aim, we chose axitinib, a small molecule inhibitor of vascular endothelial growth factor receptors 1–3, platelet-derived growth factor receptor, and stem cell growth factor receptor c-Kit [34]. This potent drug gained FDA approval for treatment of advanced renal cell carcinoma in 2012 [56] and it is still tested for other types of cancer. Moreover, axitinib was already evaluated in murine melanoma models where it has proven its efficacy either alone [36] or with combination with other therapies [35, 57] and continues to be assessed for melanoma treatment in clinical trials [58, 59].

We observed differences in effects of axitinib treatment in compared models. Although axitinib inhibited tumor growth both in classical and spheroid-plug model (Fig. 7b), the calculated necrosis factor indicated that axitinib decreases the viability of tumor cells only in spheroid-plug model (Fig. 7d). The potential of axitinib to reduce vascularization of tumors was better visible in spheroid-plug model than in classical approach (Fig. 7e, f). Given the known mechanism of axitinib action [36], it could be expected that axitinib should decrease vascularization and cause necrosis—explaining the best visible effect in spheroid-plug model. Therefore, this work not only shows the suitability of spheroid-plug model to test antiangiogenic drugs but also its advantages over the classical model in term of drug effectiveness.

Spheroid-plug model could also be used to study metastasis of tumor cells. While the frequency of metastasis is lower than in the classical model, axitinib completely abolished metastasis in spheroid-plug model. These results are in accordance with already published studies about axitinib influence on inhibiting melanoma metastasis [36].

Altogether, spheroid-plug model affects kinetics of tumor growth rate, necrosis content, vascularization of tumors, infiltration by progenitor cells, and presence of cancer cells with stem cell phenotype. These are the crucial aspects of tumor development that are investigated nowadays as a possible targets of therapeutic intervention. Without burdensome modifications of classical subcutaneous model, the spheroid-plug model allows studying tumors that develop differentially to classical subcutaneous methods regarding important tumor determinants. The spheroid-plug model could be an alternative to classical approach for antiangiogenic drug screening as it showed advantages over classical model in testing an already evaluated compound. Nevertheless, studies including antitumor therapy testing combined with comparison to clinical data are needed to further verify whether spheroid-plug model better reflects the complexity of tumor development and consequently if it is reliable for drug screening.

## Electronic supplementary material

Below is the link to the electronic supplementary material.

Fig. S1 Spheroids structure after needle transfer. (GIF 22 kb)

High-resolution image (TIFF 977 kb)

Fig. S2 Antibodies used in the flow cytometry analysis. (GIF 122 kb)

High-resolution image (TIFF 1,183 kb)

Fig. S3 The induction of cancer stem cell phenotype by in vitro spheroid culture. (A) Frequency of CXCR4^+^CD49f^+^ cells in B16 in vitro cultures. (B) Number of CXCR4^+^CD49f^−^ cells and (C) c-Kit^−^CD133^+^Sca-1^+^ cells in LLC in vitro cultures. (GIF 38 kb)

High-resolution image (TIFF 1,354 kb)

## References

[1] Kerbel RS (2003). Human tumor xenografts as predictive preclinical models for anticancer drug activity in humans: better than commonly perceived—but they can be improved. Cancer Biol Ther.

[2] Suggitt M, Bibby MC (2005). 50 years of preclinical anticancer drug screening: empirical to target-driven approaches. Clin Cancer Res.

[3] Perel P, Roberts I, Sena E, Wheble P, Briscoe C, Sandercock P (2007). Comparison of treatment effects between animal experiments and clinical trials: systematic review. BMJ.

[4] Mak IW, Evaniew N, Ghert M (2014). Lost in translation: animal models and clinical trials in cancer treatment. Am J Transl Res.

[5] Hackam DG, Redelmeier DA (2006). Translation of research evidence from animals to humans. JAMA.

[6] Vasudev NS, Reynolds AR (2014). Anti-angiogenic therapy for cancer: current progress, unresolved questions and future directions. Angiogenesis.

[7] Teicher BA (2006). Tumor models for efficacy determination. Mol Cancer Ther.

[8] Richmond A, Su Y (2008). Mouse xenograft models vs GEM models for human cancer therapeutics. Dis Model Mech.

[9] Hollingshead MG (2008). Antitumor efficacy testing in rodents. J Natl Cancer Inst.

[10] Bibby MC (2004). Orthotopic models of cancer for preclinical drug evaluation: advantages and disadvantages. Eur J Cancer.

[11] Rosenberg DW, Giardina C, Tanaka T (2009). Mouse models for the study of colon. Carcinogenesis.

[12] Singh M, Murriel CL, Johnson L (2012). Genetically engineered mouse models: closing the gap between preclinical data and trial outcomes. Cancer Res.

[13] Weber K, Bartsch U, Stocking C, Fehse B (2008). A multicolor panel of novel lentiviral “Gene Ontology” (LeGO) vectors for functional gene analysis. Mol Ther.

[14] Korff T, Augustin HG (1998). Integration of endothelial cells in multicellular spheroids prevents apoptosis and induces differentiation. J Cell Biol.

[15] Bergers G, Benjamin LE. Tumorigenesis and the angiogenic switch. Nat Rev Cancer. 2003 [cited 2014 Apr 28];3(6):401–10. Available from: http://www.ncbi.nlm.nih.gov/pubmed/12778130. 10.1038/nrc1093 12778130

[16] Jain RK. Normalization of tumor vasculature: an emerging concept in antiangiogenic therapy. Science [Internet]. 2005 Jan 7;307(5706):58–62. Available from: http://www.ncbi.nlm.nih.gov/pubmed/15637262. 10.1126/science.1104819 15637262

[17] Ahn G-O, Brown JM (2009). Role of endothelial progenitors and other bone marrow-derived cells in the development of the tumor vasculature. Angiogenesis.

[18] Weis SM, Cheresh DA (2011). Tumor angiogenesis: molecular pathways and therapeutic targets. Nat Med.

[19] Huang W-H, Chang M-C, Tsai K-S, Hung M-C, Chen H-L, Hung S-C (2013). Mesenchymal stem cells promote growth and angiogenesis of tumors in mice. Oncogene.

[20] Rafii S, Lyden D, Benezra R, Hattori K, Heissig B (2002). Vascular and haematopoietic stem cells: novel targets for anti-angiogenesis therapy?. Nat Rev Cancer.

[21] Fang S, Salven P (2011). Stem cells in tumor angiogenesis. J Mol Cell Cardiol.

[22] Wara AK, Croce K, Foo S, Sun X, Icli B, Tesmenitsky Y (2011). Bone marrow-derived CMPs and GMPs represent highly functional proangiogenic cells: implications for ischemic cardiovascular disease. Blood.

[23] Hirschhaeuser F, Menne H, Dittfeld C, West J, Mueller-Klieser W, Kunz-Schughart L a. Multicellular tumor spheroids: an underestimated tool is catching up again. J Biotechnol. Elsevier B.V.; 2010 Jul 1 [cited 2014 May 26];148(1):3–15. Available from: http://www.ncbi.nlm.nih.gov/pubmed/20097238. 10.1016/j.jbiotec.2010.01.012 20097238

[24] Robertson FM, Ogasawara MA, Ye Z, Chu K, Pickei R, Debeb BG (2010). Imaging and analysis of 3D tumor spheroids enriched for a cancer stem cell phenotype. J Biomol Screen.

[25] Liao J, Qian F, Tchabo N, Mhawech-Fauceglia P, Beck A, Qian Z (2014). Ovarian cancer spheroid cells with stem cell-like properties contribute to tumor generation, metastasis and chemotherapy resistance through hypoxia-resistant metabolism. PLoS One.

[26] Levina V, Marrangoni A, Wang T, Parikh S, Su Y, Herberman R (2010). Elimination of human lung cancer stem cells through targeting of the stem cell factor-c-kit autocrine signaling loop. Cancer Res.

[27] Kim CFB, Jackson EL, Woolfenden AE, Lawrence S, Babar I, Vogel S (2005). Identification of bronchioalveolar stem cells in normal lung and lung cancer. Cell.

[28] Monzani E, Facchetti F, Galmozzi E, Corsini E, Benetti A, Cavazzin C (2007). Melanoma contains CD133 and ABCG2 positive cells with enhanced tumourigenic potential. Eur J Cancer.

[29] Wu Y, Wu PY (2009). CD133 as a marker for cancer stem cells: progresses and concerns. Stem Cells Dev.

[30] Lee N, Barthel SR, Schatton T (2014). Melanoma stem cells and metastasis: mimicking hematopoietic cell trafficking?. Lab Investig.

[31] Zucchi I, Sanzone S, Astigiano S, Pelucchi P, Scotti M, Valsecchi V (2007). The properties of a mammary gland cancer stem cell. Proc Natl Acad Sci.

[32] Bertolini G, Roz L, Perego P, Tortoreto M, Fontanella E, Gatti L (2009). Highly tumorigenic lung cancer CD133+ cells display stem-like features and are spared by cisplatin treatment. Proc Natl Acad Sci.

[33] Nian W-Q, Chen F-L, Ao X-J, Chen Z-T (2011). CXCR4 positive cells from Lewis lung carcinoma cell line have cancer metastatic stem cell characteristics. Mol Cell Biochem.

[34] Escudier B, Gore M (2011). Axitinib for the management of metastatic renal cell carcinoma. Drugs R D.

[35] Zhang XH, Qiao EQ, Gao Z, Yuan HQ, Cai PF, Li XM (2013). Efficacy of combined axitinib with dacarbazine in a B16F1 melanoma xenograft model. Oncol Lett.

[36] Zhang X, Fang X, Gao Z, Chen W, Tao F, Cai P, et al. Axitinib, a selective inhibitor of vascular endothelial growth factor receptor, exerts an anticancer effect in melanoma through promoting antitumor immunity. Anticancer Drugs. 2014;25(2):204–11. Available from: http://www.ncbi.nlm.nih.gov/pubmed/24135499 10.1097/CAD.0000000000000033 24135499

[37] Sutherland RM, MacDonald HR, Howell RL. Multicellular spheroids: a new model target for in vitro studies of immunity to solid tumor allografts. J Natl Cancer Inst. 1977 Jun [cited 2014 May 7];58(6):1849–53. Available from: http://www.ncbi.nlm.nih.gov/pubmed/140943. 10.1093/jnci/58.6.1849 140943

[38] Culo F, Yuhas J, Ladman A. Multicellular tumour spheroids: a model for combined in vivo/in vitro assay of tumour immunity. Br J Cancer. 1980 [cited 2014 Jun 7];41(1):100–12. Available from: http://www.ncbi.nlm.nih.gov/pmc/articles/PMC2010180/. 10.1038/bjc.1980.11 PMC2010180 7362771

[39] Landry JM, Lord EM, Sutherland RM. In vivo growth of tumor cell spheroids after in vitro hyperthermia. Cancer Res. 1982 Jan [cited 2014 Jun 7];42(1):93–9. Available from: http://cancerres.aacrjournals.org/content/42/1/93.short 7032696

[40] Hanahan D, Weinberg RA (2011). Hallmarks of cancer: the next generation. Cell.

[41] Cavallo F, Offringa R, van der Burg SH, Forni G, Melief CJM (2006). Vaccination for treatment and prevention of cancer in animal models. Adv Immunol.

[42] Venanzi F, Shifrin V, Sherman M, Gabai V, Kiselev O, Komissarov A, et al. Broad-spectrum anti-tumor and anti-metastatic DNA vaccine based on p62-encoding vector. Oncotarget. 2013;4(10):1829–35. Available from: http://www.pubmedcentral.nih.gov/articlerender.fcgi?artid=3858567&tool=pmcentrez&rendertype=abstract 10.18632/oncotarget.1397 PMC3858567 24121124

[43] Gilchrist KW, Gray R, Fowble B, Tormey DC, Taylor SG (1993). Tumor necrosis is a prognostic predictor for early recurrence and death in lymph node-positive breast cancer: a 10-year follow-up study of 728 Eastern Cooperative Oncology Group patients. J Clin Oncol.

[44] Richards CH, Mohammed Z, Qayyum T, Horgan PG, McMillan DC (2011). The prognostic value of histological tumor necrosis in solid organ malignant disease: a systematic review. Future Oncol.

[45] Ferrara N, Kerbel RS (2005). Angiogenesis as a therapeutic target. Nature.

[46] Medina MÁ, Muñoz-Ch´puli R, Quesada AR (2007). Challenges of antiangiogenic cancer therapy: trials and errors, and renewed hope. J Cell Mol Med.

[47] Wu JM, Staton CA (2012). Anti-angiogenic drug discovery: lessons from the past and thoughts for the future. Expert Opin Drug Discov.

[48] Davidoff AM, Ng CYC, Brown P, Leary MA, Spurbeck WW, Zhou J (2001). Bone marrow-derived cells contribute to tumor neovasculature, and when modified to express an angiogenesis inhibitor, can restrict tumor growth in mice. Clin Cancer Res.

[49] Miletic H, Fischer Y, Litwak S, Giroglou T, Waerzeggers Y, Winkeler A (2007). Bystander killing of malignant glioma by bone marrow-derived tumor-infiltrating progenitor cells expressing a suicide gene. Mol Ther.

[50] Studeny M, Marini FC, Dembinski JL, Zompetta C, Cabreira-Hansen M, Bekele BN (2004). Mesenchymal stem cells: potential precursors for tumor stroma and targeted-delivery vehicles for anticancer agents. J Natl Cancer Inst.

[51] Collet G, Lamerant-Fayel N, Tertil M, El Hafny-Rahbi B, Stepniewski J, Guichard A (2014). Hypoxia-regulated overexpression of soluble VEGFR2 controls angiogenesis and inhibits tumor growth. Mol Cancer Ther.

[52] Kim M, Koh YJ, Kim KE, Koh BI, Nam D-H, Alitalo K (2010). CXCR4 signaling regulates metastasis of chemoresistant melanoma cells by a lymphatic metastatic niche. Cancer Res.

[53] Sutherland RM (1988). Cell and environment interactions in tumor microregions: the multicell spheroid model. Science.

[54] Mehta G, Hsiao AY, Ingram M, Luker GD, Takayama S (2012). Opportunities and challenges for use of tumor spheroids as models to test drug delivery and efficacy. J Control Release.

[55] Korff T, Krauss T, Augustin HG (2004). Three-dimensional spheroidal culture of cytotrophoblast cells mimics the phenotype and differentiation of cytotrophoblasts from normal and preeclamptic pregnancies. Exp Cell Res.

[56] U.S. Food and Drug Administration. Axitinib. [cited 2015 Aug 23]. Available from: http://www.fda.gov/Drugs/InformationOnDrugs/ApprovedDrugs/ucm289439.htm

[57] Bose A, Lowe DB, Rao A, Storkus WJ. Combined vaccine + axitinib therapy yields superior antitumor efficacy in a murine melanoma model. Melanoma Research. 2012. p. 236–43. 10.1097/CMR.0b013e3283538293 PMC3340498 22504156

[58] Fruehauf J, Lutzky J, McDermott D, Brown CK, Meric J-B, Rosbrook B (2011). Multicenter, phase II study of axitinib, a selective second-generation Inhibitor of vascular endothelial growth factor receptors 1, 2, and 3, in patients with metastatic melanoma. Clin Cancer Res.

[59] Algazi AP, Cha E, Ortiz-Urda SM, McCalmont T, Bastian BC, Hwang J, et al. The combination of axitinib followed by paclitaxel/carboplatin yields extended survival in advanced BRAF wild-type melanoma: results of a clinical/correlative prospective phase II clinical trial. Br J Cancer [Internet]. 2015; Available from: http://www.nature.com/doifinder/10.1038/bjc.2014.541. 10.1038/bjc.2014.541 PMC4402449 25867272

