# Supplementary figures and images for: Spheroid-plug model as a tool to study tumor development, angiogenesis, and heterogeneity in vivo
Source: Tumour Biol. 2015 Sep 18;37(2):2481–96. doi: 10.1007/s13277-015-4065-z (PMC4842223; doi:10.1007/s13277-015-4065-z)

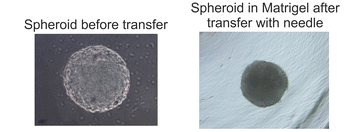

Supplement: Supplementary file 1 — Spheroids structure after needle transfer. (GIF 22 kb) [file 13277_2015_4065_Fig10_ESM.gif]

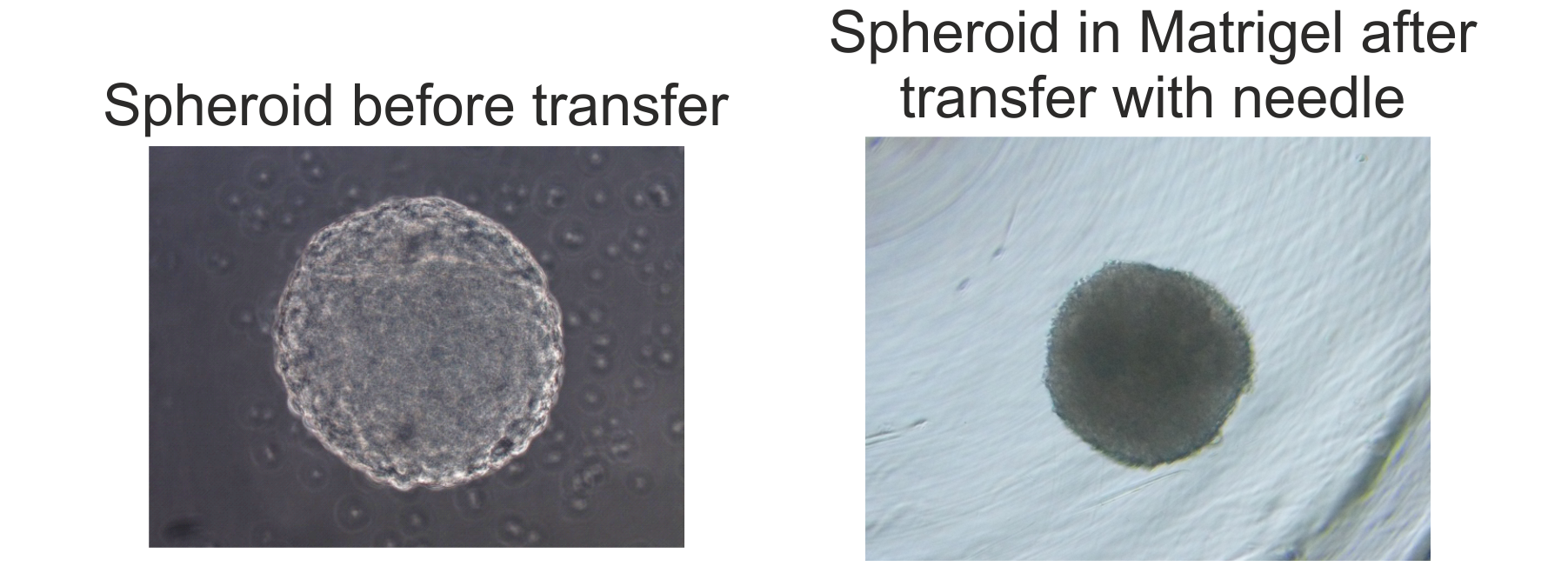

Supplement: Supplementary file 2 — High-resolution image (TIFF 977 kb) [file 13277_2015_4065_MOESM1_ESM.tif]

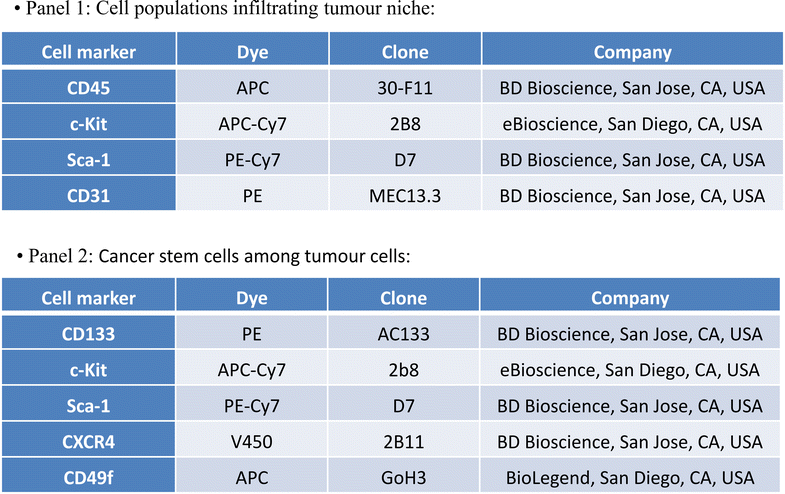

Supplement: Supplementary file 3 — Antibodies used in the flow cytometry analysis. (GIF 122 kb) [file 13277_2015_4065_Fig11_ESM.gif]

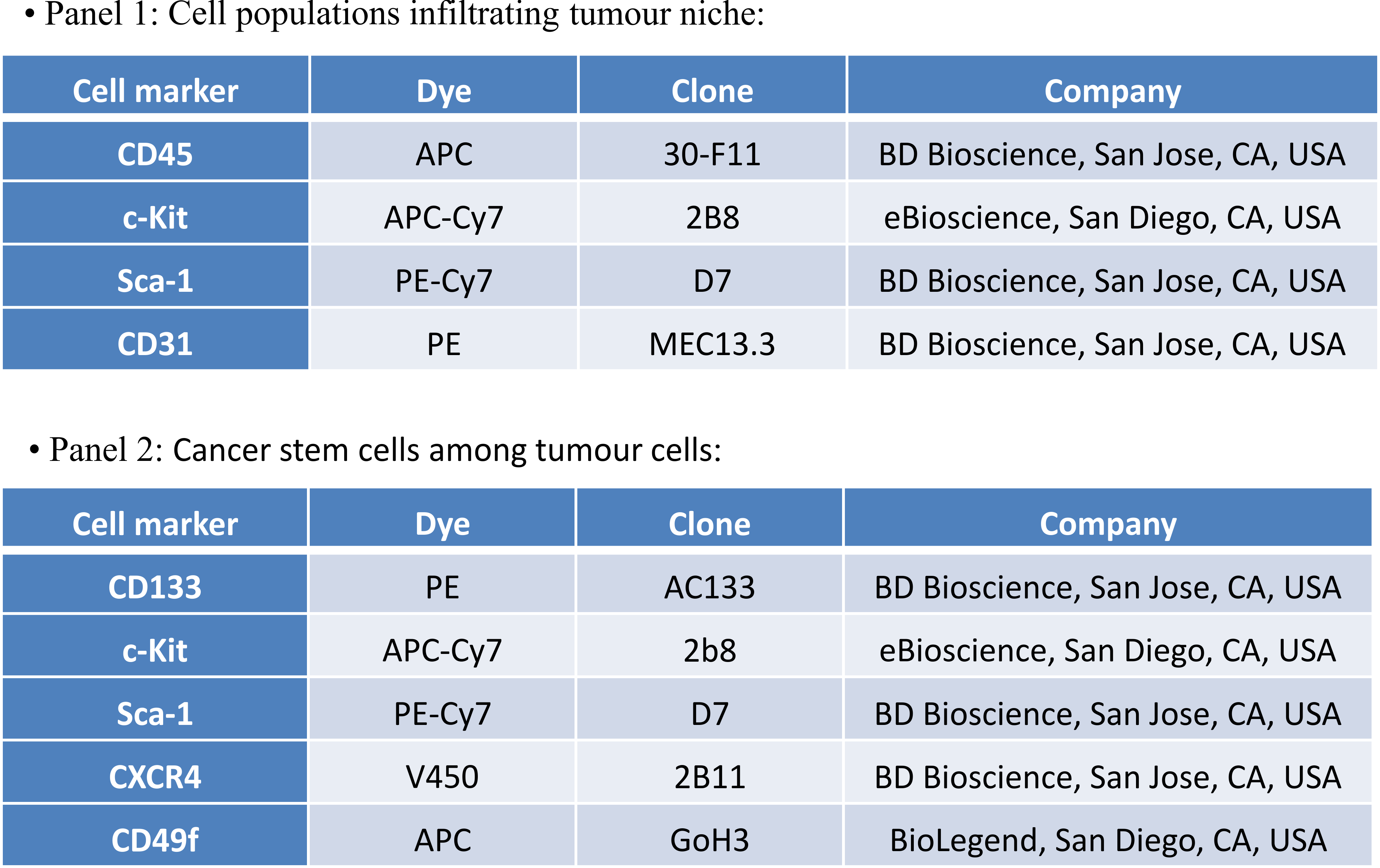

Supplement: Supplementary file 4 — High-resolution image (TIFF 1,183 kb) [file 13277_2015_4065_MOESM2_ESM.tif]

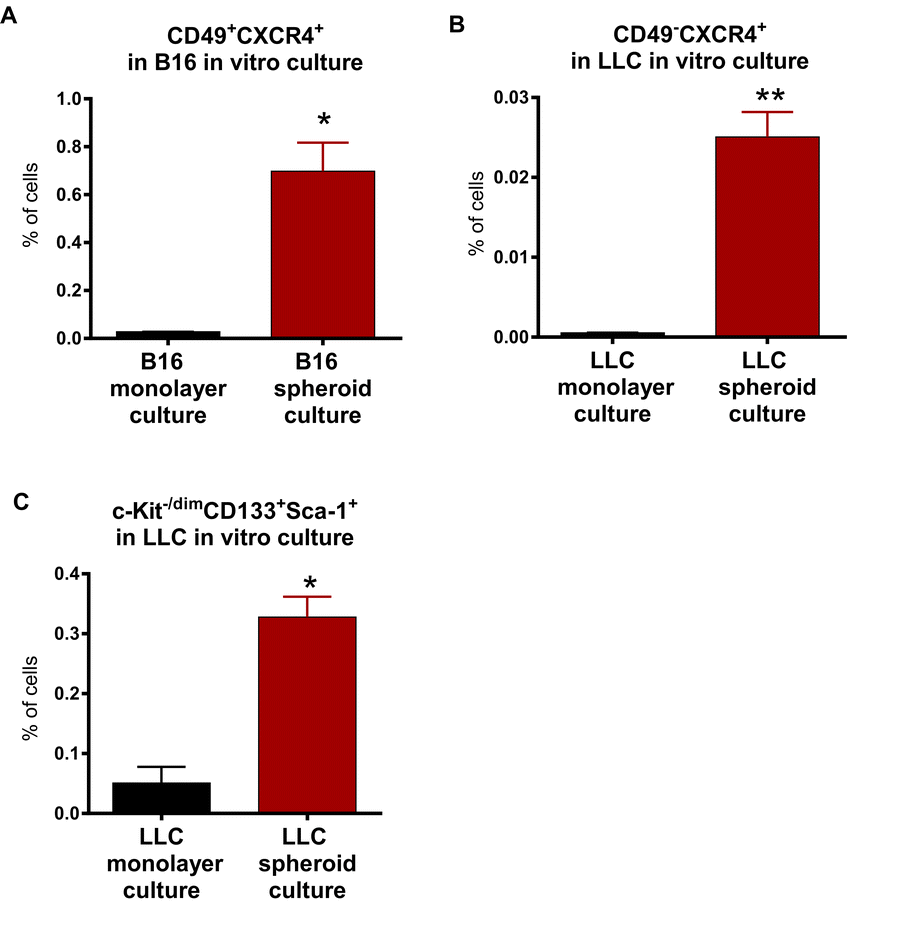

Supplement: Supplementary file 5 — The induction of cancer stem cell phenotype by in vitro spheroid culture. (A) Frequency of CXCR4+CD49f+ cells in B16 in vitro cultures. (B) Number of CXCR4+CD49f− cells and (C) c-Kit−CD133+Sca-1+ cells in LLC in vitro cultures. (GIF 38 kb) [file 13277_2015_4065_Fig12_ESM.gif]

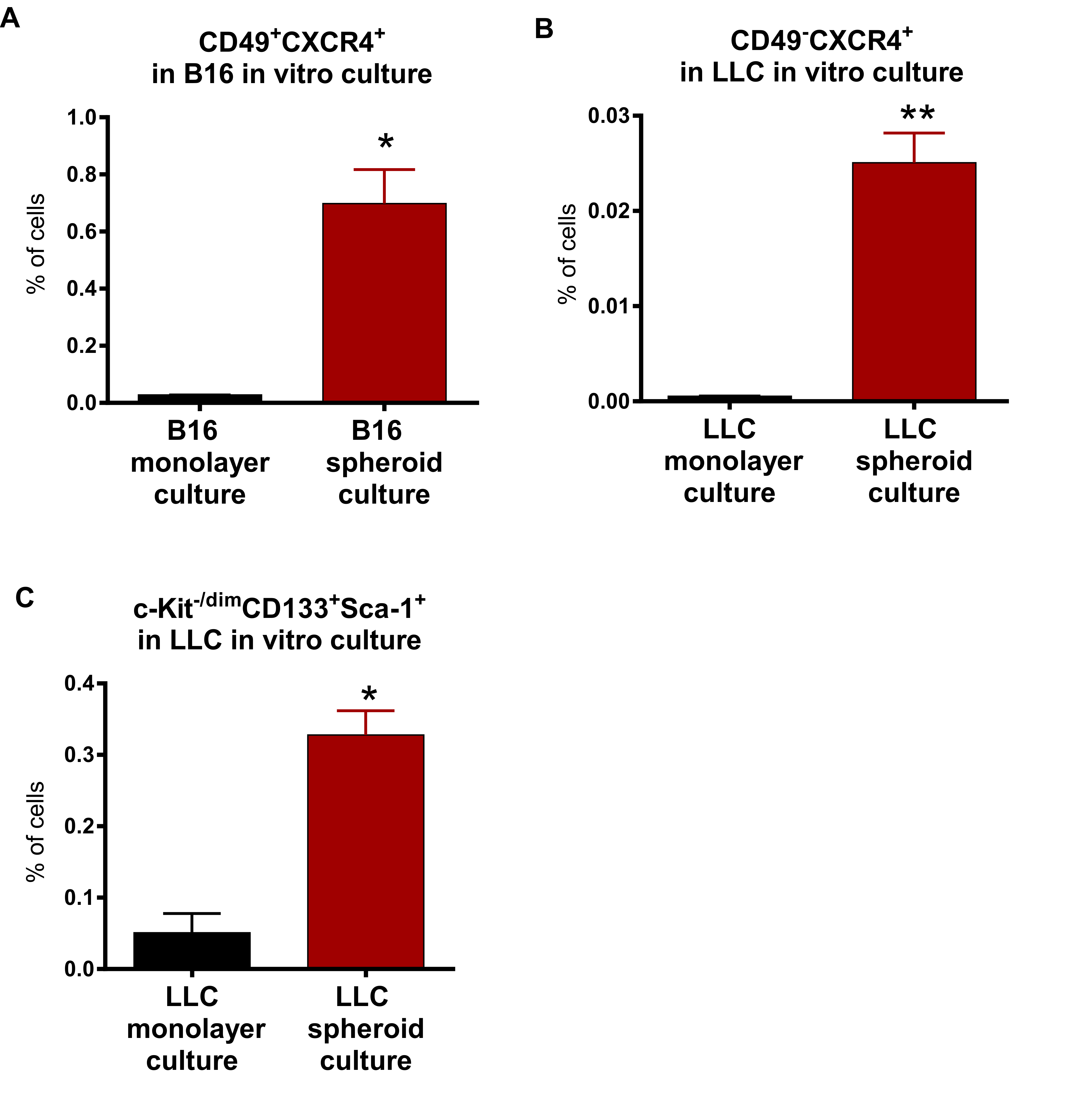

Supplement: Supplementary file 6 — High-resolution image (TIFF 1,354 kb) [file 13277_2015_4065_MOESM3_ESM.tif]
